# Application of adipose-derived mesenchymal stem cells in an *in vivo* model of peripheral nerve damage

**DOI:** 10.3389/fncel.2022.992221

**Published:** 2022-09-08

**Authors:** Elsa González-Cubero, María Luisa González-Fernández, María Rodríguez-Díaz, Marta Palomo-Irigoyen, Ashwin Woodhoo, Vega Villar-Suárez

**Affiliations:** ^1^Department of Anatomy, Faculty of Veterinary Sciences, University of León-Universidad de León, León, Spain; ^2^Center for Cooperative Research in Biosciences (CIC bioGUNE), Basque Research and Technology Alliance (BRTA), Bizkaia Technology Park, Derio, Spain; ^3^Genes and Disease Group, Department of Dermatology, Medical University of Vienna, Anna Spiegel Center of Translational Research, Vienna, Austria; ^4^IKERBASQUE, Basque Foundation for Science, Bilbao, Spain; ^5^Gene Regulatory Control in Disease Group, Center for Research in Molecular Medicine and Chronic Diseases (CIMUS), Health Research Institute of Santiago de Compostela (IDIS), University of Santiago de Compostela, Santiago de Compostela, Spain; ^6^Institute of Biomedicine (IBIOMED), University of León-Universidad de León, León, Spain

**Keywords:** adipose tissue derived-mesenchymal stem cells, Schwann cells, peripheral neuropathy, conditioned medium, peripheral nerve regeneration, nerve regeneration using mesenchymal stem cells

## Abstract

**Background:**

Neuropathic pain is one of the most difficult to treat chronic pain syndromes. It has significant effects on patients’ quality of life and substantially adds to the burden of direct and indirect medical costs. There is a critical need to improve therapies for peripheral nerve regeneration. The aim of this study is to address this issue by performing a detailed analysis of the therapeutic benefits of two treatment options: adipose tissue derived-mesenchymal stem cells (ASCs) and ASC-conditioned medium (CM).

**Methods:**

To this end, we used an *in vivo* rat sciatic nerve damage model to investigate the molecular mechanisms involved in the myelinating capacity of ASCs and CM. Furthermore, effect of TNF and CM on Schwann cells (SCs) was evaluated. For our *in vivo* model, biomaterial surgical implants containing TNF were used to induce peripheral neuropathy in rats. Damaged nerves were also treated with either ASCs or CM and molecular methods were used to collect evidence of nerve regeneration. Post-operatively, rats were subjected to walking track analysis and their sciatic functional index was evaluated. Morphological data was gathered through transmission electron microscopy (TEM) of sciatic nerves harvested from the experimental rats. We also evaluated the effect of TNF on Schwann cells (SCs) *in vitro*. Genes and their correspondent proteins associated with nerve regeneration were analyzed by qPCR, western blot, and confocal microscopy.

**Results:**

Our data suggests that both ASCs and CM are potentially beneficial treatments for promoting myelination and axonal regeneration. After TNF-induced nerve damage we observed an upregulation of c-Jun along with a downregulation of Krox-20 myelin-associated transcription factor. However, when CM was added to TNF-treated nerves the opposite effect occurred and also resulted in increased expression of myelin-related genes and their corresponding proteins.

**Conclusion:**

Findings from our *in vivo* model showed that both ASCs and CM aided the regeneration of axonal myelin sheaths and the remodeling of peripheral nerve morphology.

## Introduction

Currently, neuropathic pain (NP) affects millions of individuals globally and is an underappreciated socioeconomic health issue (McDermott et al., 2006; Carrasco et al., 2018). The International Association for the Study of Pain has recently redefined it as “pain caused by lesion or disease of the somatosensory system” and it may manifest in a variety of conditions. There are numerous systemic, metabolic, and toxic causes of peripheral neuropathy. The most frequent diseases are hypothyroidism, diabetes mellitus, and dietary deficits. The cause of the neuropathy is complex, involving a cascade of molecular components which simultaneously affect the inflammatory process, the axonal degenerative component, and the demyelinating impairment. However, not all these molecular factors respond to the same molecular signal. Increasing evidence suggests that inflammation and mitochondrial dysfunction brought on by oxidative and nitrosative stress serve as the physiopathological triggering for the development of degenerative nerve diseases (McDermott et al., 2006; O’Connor, 2009).

Activation of the inflammatory cascade, proinflammatory cytokine upregulation, and neuroimmune communication pathways are all implicated in peripheral neuropathy (Sandireddy et al., 2014; Castelli et al., 2016) and contribute to structural and functional nerve damage. In this way, the roles of immunological and pro-inflammatory mediators (e.g., interleukins, TNF, complement components, ATP, and chemokines) in the processes of peripheral and central neuropathic pain are a particular area of interest (Marchand et al., 2005; McMahon et al., 2005; Campbell and Meyer, 2006; Moalem and Tracey, 2006). Other research has shown that regardless of the severity and mode of nerve damage, post-injury inflammation is a common complication and can cause a variety of phenomenon which are all implicated in neuropathic pain, specifically, mast cell degranulation (Olsson, 1967) and recruitment of both macrophages (Perry et al., 1987) and polymorphonuclear neutrophils (Daemen et al., 1998). Several studies have pointed to other factors crucial to the development of neuropathic pain, for instance, the specific inflammatory microenvironment (Sommer et al., 1993) and the release of pro-inflammatory mediators (Frisén et al., 1993). Furthermore, in animal models, TNF (Wagner and Myers, 1996a,b), interleukin-1 (IL-1) (Sweitzer et al., 1999; Sommer and Kress, 2004; Zelenka et al., 2005), and interleukin-6 (IL-6) (DeLeo et al., 1996; Arruda et al., 1998) have all been linked to the development of neuropathic pain.

An emerging area of neuropathic pain research concerns the role of Schwann cells (SCs), the main glial cells of the peripheral nervous system (PNS), one of their functions is the production of the myelin sheath that surrounds neuronal axons (Bunge, 1994; Jessen and Mirsky, 2000). SCs have a well-coordinated differentiation process, and differentiated SCs create a multi-spiraled extension of the plasma membrane to allow saltatory conduction of the nerve impulse (Jessen and Mirsky, 2005).

Myelin degradation termed demyelination of peripheral nerves has a wide range of causes, including genetic or acquired illness, toxicity, or nerve injury which disrupt the action of SCs or alter the nerve cell environment, and leads to a process known as Wallerian degeneration in the distal side of the lesion (Hall, 1986; Krekoski et al., 2001). This process is required for peripheral nerve regeneration and involves the SC demyelination and conversion into repair SCs prior to nerve regeneration. Recent research has also shown that, after nerve injury, SCs are not only able to summon macrophages for myelin phagocytosis but can also digest their own myelin (Hirata and Kawabuchi, 2002; Gomez-Sanchez et al., 2015). The intrinsic potential for myelin breakdown shown by SCs must be tightly controlled in order to maintain healthy myelin and optimal nerve function. After nerve injury, SCs dedifferentiate to repair SCs, which form regeneration tracks called bands of Bungner, demonstrating the extraordinary phenotypic plasticity of SCs (Arthur-Farraj et al., 2012; BrosiusLutz and Barres, 2014; Jessen et al., 2015). However, there is a clear need for improvements in the clinical treatment of peripheral nerve damage (Taylor et al., 2008; Zheng et al., 2018). Of particular importance are therapies aimed to promote peripheral nerve repair following injury, and especially, those that speed up axonal nerve regeneration (Taylor et al., 2008; Zhao et al., 2014; Zheng et al., 2018; Beris et al., 2019). Multipotent stromal cells or mesenchymal stem cells (MSCs), particularly adipose tissue derived-mesenchymal stem cells (ASCs), are recognized to have potential in a wide range of regenerative therapies (Lavoie and Rosu-Myles, 2013; Nordberg and Loboa, 2015) including treatment of neuropathic pain (Tomita et al., 2012). Their immunoregulatory behavior and gene expression characteristics are thought to be comparable to those of bone marrow-derived stromal cells (BMSCs). ASCs also have excellent capacity of differentiation and have been shown to have neurotrophic characteristics (Wosnitza et al., 2007; Mantovani et al., 2010; Levi et al., 2013) and, in addition, ASCs have a higher production and proliferation potential than BMSCs (Gimble et al., 2007; Yoshimura et al., 2007; Liao et al., 2010). Transdifferentiation has been demonstrated in MSCs (Abderrahim-Ferkoune et al., 2004) which can differentiate into various non-mesenchymal lineages, such as astrocytes (Kopen et al., 1999), myocardium (Pittenger and Martin, 2004), endothelial cells (Oswald et al., 2004), neurons (Deng et al., 2001), and myelinating cells of the PNS (Tohill et al., 2004).

MSCs are known to release anti-inflammatory and anti-apoptotic chemicals, as well as trophic factors, which variously support axonal development, immunomodulation, angiogenesis, remyelination, and protect damaged nerve cells against apoptotic cell death (Lavoie and Rosu-Myles, 2013). Transplanted MSCs can not only differentiate into neurons and endothelial cells, but also produce a wide spectrum of physiologically active substances and extracellular vesicles (EVs), which together are known as MSC secretome or conditioned medium (CM).

To trigger responses from resident cells, MSCs can release strong combinations of trophic factors that modify the molecular structure of the environment. This “paracrine hypothesis” is now generally accepted as the guiding principle underlying MSC-based therapy. Evidence for this hypothesis lies in the finding that MSC secretome can considerably reduce neuroinflammation and restore the balance between inflammatory cytokines and signaling molecules (Brini et al., 2017; Mukhamedshina et al., 2019). Indeed, there is mounting evidence that one of the principal ways in which MSCs promote regeneration of damaged tissue is through the secretion of exosomes and growth factors (Lamichhane et al., 2015; Nakamura et al., 2015). It has also been demonstrated that ASCs have significant immunosuppressive effects and inhibit both effector T-cell and inflammatory responses (da Silva Meirelles et al., 2009; Kapur and Katz, 2013) in allogenic lymphocytes. Many other studies further support the paracrine notion and MSC therapy is increasingly based on the substances produced by MSCs (secretome or CM) rather than their differentiation ability (Stoltz et al., 2015).

Here, we compared the therapeutic effects of ASC-CM versus ASCs. We examined the pro-myelinating effect of both CM and ASCs in an *in vivo* rat model of sciatic nerve damage. We used TNF to induce neuropathy in the sciatic nerves of rats *in vivo* following treatment of either CM or ASCs to observe the regeneration capacity of each treatment. After treatment, rats were subjected to walking track analysis to evaluate their sciatic nerve functional index and a morphological analysis of the rats’ sciatic nerves was completed. We showed that the TNF induced nerve injury is counteracted by both CM and ASCs, but the effect was more prominent with CM. This appears to be due to the fact that CM has a higher potential to induce remyelination in damaged axons. We also evaluated the effect of TNF on SCs *in vitro*. Overall, the aim of this study is to observe the ASCs and CM nerve regenerative effects and preliminarily clarified the mechanism of this effect through molecular biological methods.

## Materials and methods

### Animals

The protocols for this experimental study comply with the guidelines of the council of the European Union (86/609/EU) and we have followed Spanish regulations (BOE 67/8509-12, 1998) for the use of laboratory animals. All experiments were performed in accordance with the recommendations of the Spanish Guide for the Care and Use of Laboratory Animals, and the standards set out by the International Animal Care and Use Committee. All procedures were performed following the ethical guidelines of the Biosafety and Welfare Committee of the University of León and Junta de Castilla y León (OEBA).

We used thirty female Wistar rats (Rattus Norvegicus) aged between 5 and 10 months and weighing between 300 and 400 grams. The rats were kept under conventional housing conditions (22 ± 2°C, 55 ± 10% humidity, 12-h day/night cycle) and fed a standard diet with *ad libitum* access to food and water. All possible efforts were made to minimize animal suffering and the number of animals used.

### Cell culture

#### Schwann cell isolation and purification

Schwann cells (SCs) were isolated from the sciatic nerves and brachial plexus of 3–5 postnatal day (PND) Wistar rats as described in Irigoyen et al. (2018). Briefly, tissues were surgically removed and placed in ice cold Leibovitz’s L15 medium (Gibco^®^) with antibiotic/antimycotic solution (Fisher Scientific^®^). All procedures took place under sterile conditions.

After the removal of epineural sheath, the nerve tissues were subjected to enzymatic digestion for 35 min in ambient conditions of 37°C, 5% CO_2_ and 95% humidity in a solution consisting of (per rat): 100 μl of 0.25% trypsin and 100 μl of 0.4% collagenase mix. This was followed by trituration. An equal volume of DMEM with 10% fetal bovine serum (FBS) was then added to the cell suspension, and this was centrifuged for 10 min at 1,000 rpm at 4°C. The resulting pellet was resuspended in DMEM with 10% FBS supplemented with 10^–3^ M AraC and cultured for 3 days on 35 mm PDL and laminin-coated tissue culture dishes.

Reverse immunopanning was then carried out to purify the SCs culture by removing any remaining fibroblasts. In brief, 90 mm petri dishes (Falcon^®^) were coated with rabbit anti-mouse IgG (Dako^®^) (7 mL 50 mM Tris pH 9.5 and 50 μL IgG per dish) and Thy 1.1 antibody (4 mL OX-7 supernatant, 2 mL L15 medium and 400 μL 35% BSA per dish). After 3 days of treatment with AraC, Schwann cells were then trypsinized, centrifuged and subsequently resuspended in 10 mL DMEM containing 10% FBS. The cell suspension was then incubated for 20 min under conditions of gentle shaking at 37 °C, 5% CO_2_ and 95% humidity on Thy1.1-coated dishes. The purified SC culture was pelleted by centrifugation and resuspended in expansion medium* with 0.5% FBS, 10 ng/mL neuregulin (NRG1, R&D Systems^®^) and 2 μM forskolin (Calbiochem^®^) and cultured on PDL/laminin-coated dishes. Media was replaced every 3 days until the cells were 80% confluent.

* DMEM/F12 (Gibco^®^) with: SATO [(100 μg/mL bovine serum albumin (BSA; Sigma^®^), 100 μg/mL transferrin powder (Sigma^®^), 16 μg/mL Putrescine; 60 ng/mL progesterone (Sigma^®^) and 40 ng/mL sodium selenite (Sigma^®^) in Neurobasal medium (Gibco^®^)]; 1X B27 supplement; 0.101 mg/mL of 3.3′,5-Triiodo-L-thyronine sodium salt (T3, Sigma^®^); 10 mM Insulin; 1% L-glutamine and 1% A/A.

#### Schwann cell differentiation

Purified and expanded SCs were treated with 4 mM N6,2′-*O-*Dibutyryladenosine3′,5′-cyclic monophosphate sodium salt (dB-cAMP) for 48 h to induce up regulation of myelin differentiation markers after which they were seeded and then cultured overnight in starvation medium.^**^

^**^ DMEM/F12 (Gibco^®^) with SATO [(100 μg/mL bovine serum albumin (BSA; Sigma^®^), 100 μg/mL transferrin powder (Sigma^®^), 16 μg/mL Putrescine; 60 ng/mL progesterone (Sigma^®^) and 40 ng/mL sodium selenite (Sigma^®^) in Neurobasal medium (Gibco^®^)], 1X B27 supplement; 0.101 mg/mL of 3.3′,5-Triiodo-L-thyronine sodium salt (T3, Sigma^®^), 10 mM Insulin, 1% L-glutamine and 1% A/A) supplemented with 0.5% FBS.

#### Adipose-derived mesenchymal stem cells isolation and culture

Rat abdominal and inguinal adipose tissue was harvested to extract ASCs. Approximately 5 grams of fat was obtained, and this was digested at 37°C for 1 h with 0.075% type I collagenase (Sigma^®^). The supernatant was removed after centrifugation, and the pellet was resuspended in phosphate buffered saline (PBS, Sigma^®^) then filtered (100 m Nylon Mesh, Fisherbrand^®^). Isolated cells were grown in monolayer cultures in complete medium (DMEMc)^***^ at 37°C in a humid atmosphere containing 5% CO_2_. The medium was replaced every 2–3 days and adherent cells were cultured until they reached 80% confluence.

^***^Low-glucose Dulbecco’s modified Eagle’s medium (DMEM, Hyclone^®^) supplemented with 10% (v/v) FBS (Hyclone^®^) and 1% (v/v) penicillin/streptomycin (Gibco^®^).

#### Biomaterial-stem cell implants

A collagen repair patch (Zimmer^®^) was used in the assay for the *in vivo* model of sciatic nerve damage. ASCs were re-suspended in the DMEMc, then 100 μL of cell suspension (0.5 × 10^6^ cells) was seeded onto the collagen-elastin scaffold (0.5 cm^2^) in a 24-well plate. The cells were cultured for 2 h in a humidified atmosphere containing 5% CO_2_ at 37°C, after which 900 μL of DMEMc were added slowly into each well. The ASCs were then cultured for 1 week before implantation.

#### Conditioned medium collection

Passage 2 expanded ASCs (∼1 × 10^6^ cells) were cultured in DMEMc to approximately 80% confluency. To avoid possible contamination by factors present in FBS, cells were supplemented with serum-free DMEM (Hyclone^®^) with 1% penicillin/streptomycin (Hyclone^®^) and supernatants were collected (conditioned medium) after 24 h.

Supernatants were processed to make a concentrated conditioned medium. For this, 15 mL of the supernatant was transferred to an Amicon^®^ Ultra-15 10K filter and centrifuged in an Allegra^®^ X-15R centrifuge at 4,000 *g* at 4°C for 30 min. This produced 500 μL of concentrated CM. The protein concentrations of the CM and the concentrated CM were measured using a Micro BCA Protein Assay Kit, using BSA as a standard and following the manufacturer’s instructions. Total protein concentration measured in CM was 50 μg/mL and 100 μg/mL in concentrated CM.

Experiments *in vitro* with either CM or concentrated CM were performed with equal amounts of protein. Due to the fact that concentrated CM gave the best results *in vitro*, concentrated CM was used in all the *ex vivo* and *in vivo* experiments.

### *In vitro* model of Schwann cell demyelination

To provide our *in vitro* model of inflammation, *in vitro* myelinated primary rat SCs (Irigoyen et al., 2018) were plated into 6-well culture dishes. All samples were cultured in serum-free DMEM (Hyclone^®^) with 1% penicillin and streptomycin (Hyclone^®^). Samples were treated as follows:

• Starvation medium control: no other treatment
• Myelination control: pre-treated with 4 mM of dibutyryl cyclic adenosine monophosphate (dbcAMP) over a period of 48 h to induce up regulation of myelination.
• Inflammation group: Treated with 25 ng/mL TNF (Gibco^®^) only after pre-treatment with 4 mM of DB-cAMP over a period of 48 h to induce up regulation of myelination.
• CM groups: Treated with 25 ng/mL TNF (Gibco^®^) and either concentrated CM or CM after pre-treatment with 4 mM of DB-cAMP for 48 h to induce up regulation of myelination.

The SCs were then collected after 24, 48 and 72 h and analyzed using western blotting.

### *Ex vivo* model of sciatic nerve damage

The sciatic nerves of adult rats were extracted and cleaned by removing the connective tissue. During this process the nerves were kept in a 35-mm cell culture dishes containing cold PBS. Extracted sciatic nerves were then divided into 3 to 4 mm segments. Nerve fragments were transferred to a 24-well plate with three to four nerve segments per well. All samples were cultured with DMEM (Hyclone^®^) with 1% penicillin/streptomycin (Hyclone^®^) and divided into four treatment groups as follows:

• Group 1 (control group): no further treatment
• Group 2 (control-CM): treated with CM.
• Group 3 (TNF group): treated with 25 ng/mL TNF (Gibco^®^).
• Group 4 (TNF-CM group): treated with 25 ng/mL TNF (Gibco^®^) and CM.

The sciatic nerve explants were cultured for 1, 3 and 7 days and harvested to analysis using western blot, confocal microscopy, and qPCR.

### Protein analysis

#### Total protein extraction and quantification

SCs from all experimental groups of the *in vitro* model, plated in 6 well-culture dishes, were scraped in 200 μL of RIPA lysis buffer [500 mL stock solution: 1.6 mM NaH2PO4 (Merck^®^), 8.4 mM Na2HPO4 (Merck^®^), 0.1% TritonX-100 (VWR^®^), 0.1 M NaCl (Ambion^®^), 0.1% sodium dodecyl sulfate (SDS; Fisher Scientific^®^) and ddH2O] supplemented with sodium deoxycholate (Merck^®^), 1 mM sodium fluoride and 1X protease and phosphatase inhibitor cocktails (Roche^®^). Protein concentration was quantified using a Bicinchoninic acid (BCA) protein assay (Bio-Rad^®^) in a SpectraMax microplate reader (bioNova científica^®^).

Using a Swann-Morton^®^ scalpel, frozen sciatic nerve segments (5 mm) were chopped on dry ice and resuspended in 150 L of RIPA lysis buffer [500 mL stock solution: 1.6 mM NaH2PO4 (Merck), 8.4 mM Na2HPO4 (Merck^®^), 0.1% Triton X-100 (VWR^®^), 0.1 M NaCl (Ambion^®^), 0.1% sodium dodecyl sulfate (SDS; Fisher Scientific^®^) and ddH2O] supplemented with sodium deoxycholate (Merck^®^), 1 mM sodium fluoride with both 1X protease and 1X phosphatase inhibitor cocktails (Roche^®^). Samples were then homogenized in a Precellys 24 tissue grinder (Bertin Technologies^®^).

#### Western blot analysis

Samples containing 10 μg total protein were denatured by boiling in a mixture of 5X loading buffer [250 mM Tris–HCl (Sigma^®^) pH 6.8, 500 mM β-mercaptoethanol (Sigma^®^), 50% glycerol (Sigma^®^), 10% SDS (Sigma^®^), and bromophenol blue (Sigma^®^)] and H_2_O. Proteins were resolved by SDS-PAGE in 8%, 11% or 15% acrylamide gels, using a Mini-PROTEAN Electrophoresis System (Bio-Rad^®^). Fractionated proteins were transferred onto nitrocellulose membranes by electroblotting using a Mini *Trans-*Blot cell (Bio-Rad^®^). Membranes were blocked with 5% non-fat dry milk in 1X Tris Buffer Saline (TBS) [50 mM Tris, 150 mM NaCl (Sigma^®^), pH 8.0) containing 0.1% Tween-20 (Sigma^®^) (TBST-0.1%)] and incubated with primary antibodies overnight at 4°C (Table 1). Horseradish peroxidase-conjugated antibodies were used as secondary antibodies to detect immunoreactive protein bands by Western Lightning Enhanced Chemiluminiscence (ECL) Reagent (PerkinElmer^®^) and exposed to X-ray films (Fujifilm^®^) in a Curix 60 Developer (Agfa^®^).

**TABLE 1:** Primary antibodies used for protein detection.

Target protein	Dilution
β-Actin (Abcam^®^)	1:5,000
Krox20 (Abcam^®^)	1:1,000
MPZ (Abcam^®^)	1:1,000
MBP (Abcam^®^)	1:1,000

Target proteins of the antibodies, used dilution, host and company are specified.

### Confocal microscopy

Sciatic nerves from cultured explants were harvested at 1, 3 and 7 days and washed with 1X PBS before the analysis. In brief, nerves were mounted on microscope slides, stretched out and fixed in 2% paraformaldehyde for 10 min. Samples were bathed in cold methanol (−20°C) for 10 min then blocked using an antibody diluent solution (ADS; [Abcam^®^], composed of PBS containing 5% goat serum and 1% BSA) containing 0.3% Triton X-100 (Sigma^®^). After this process, samples underwent MPZ antibody incubation (1:100 dilution in ADS) overnight at 4°C. Next day, samples were incubated with Alexa-488 secondary antibody (1:100 dilution; Invitrogen^®^) and 50 ng/mL DAPI (Sigma^®^) nuclear stain (protected from light) for 30 min at RT. Samples were then mounted in Dako fluorescence mounting medium (Dako^®^) and analyzed using a confocal microscopy (Zeiss^®^).

### qPCR analysis

For qPCR analysis total RNA was isolated from a) sciatic nerve explants using TRIzol reagent (Sigma^®^) and b) from cultured SCs using a QIAshredder homogenizer (Qiagen^®^) and an AllPrep DNA/RNA mini kit (Qiagen^®^), following the manufacturer’s instructions in all cases. RNA concentration was determined using an RNA BR Qubit^®^ assay kit (Life Technologies^®^) in a Qubit^®^ 2.0 fluorometer (Life Technologies^®^) according to the manufacturer’s instructions. Total RNA was reverse transcribed using a high-capacity cDNA reverse transcription kit (Applied Biosystems^®^) following manufacturer’s instructions. Reactions were carried out in a Thermocycler (Biometra^®^).

qPCR was performed (following manufacturer’s instructions) using Power SYBR™ Green PCR Master Mix 2 × (Applied Biosystems^®^) in a total volume of 20 μL in a StepOne real-time PCR system (Applied Biosystems^®^). Quantification was performed using the 2–ΔΔCt method and normalized using *Gapdh* mRNA as a standard. Optimal PCR conditions for the primers were found to be 40 cycles with a melting temperature of 60°C, and 30 s for each step. Specific primers (Table 2) were designed using the Primer-BLAST tool and synthesized by Sigma-Aldrich^®^.

**TABLE 2:** Rat primers used for qPCR.

Target gene	Forward (5′- > 3′)	Reverse (5′- > 3′)
*Gapdh*	GTGCAGTGCCAGCCTCGTCTCATAG	TTTGTCACAAGAGAAGGCAGCCCT
*c-Jun*	GCCACCGAGACCGTAAAGAA	TAGCACTCGCCCAACTTCAG
*Krox20*	TGGGCGAGGGGACACACTGA	GCCTGGGATCCGGCTGTTGG
*Mpz*	AGGACTCCTCGAAGCGCGGG	GGCAGCTTTGGTGCTTCGGC
*Periaxin*	CTGGGGAAGCGGGGTTGCTG	GGCTTGCTCTCAGCCCCTGC

### *In vivo* model of sciatic nerve injury

#### Surgical procedure

**FIGURE 1:**
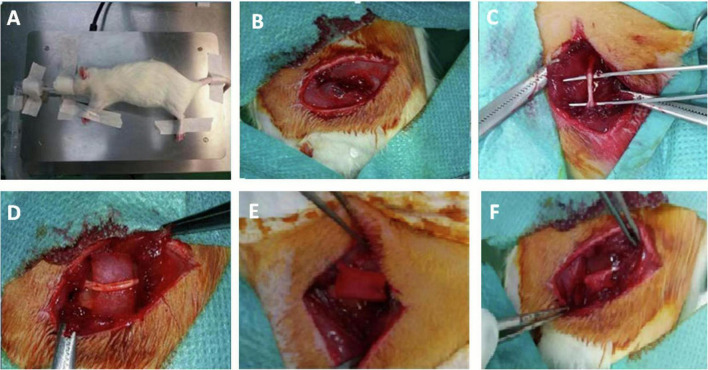
Surgical procedure. **(A)** Rat in lateral decubitus. A mask was used for the administration of the anesthetic. **(B)** Incision to the biceps femoris muscle. **(C)** Exposed sciatic nerve. **(D)** Biomaterial placed below the sciatic nerve. **(E)** Biomaterial sleeve around the sciatic nerve. **(F)** Biomaterial sleeve attached to the muscle.

Animals were deeply anesthetized using inhalation of anesthesia for all experimental procedures. For that, rats were placed in an induction chamber with isoflurane (IsoFlo^®^) and the oxygen flow was adjusted to 0.8–1.5 L/min, and the vaporizer (Isotec^®^) to deliver a concentration of 3–5%. For maintenance, a mask was secured around the rat’s snout and the flowmeter adjusted to 400–800 mL/min and the vaporizer to 2–2.5% (Figure 1A). It was necessary to apply an ophthalmic gel (Siccafluid^®^) or serum to avoid corneal injury. All surgeries were performed by the same surgeon.

The rats were divided into four experimental groups and each group received a different surgical implant of biomaterial to the sciatic nerve. The groups and their treatments were as follows:

• Biomaterial control group (1): biomaterial implant containing DMEM without cellular content to test whether the biomaterial alone had any effect.
• TNF group (2): biomaterial implant containing TNF (25 ng/μL) (Gibco^®^).
• ASC group (3): biomaterial implant containing TNF (25 ng/μL) (Gibco^®^) and ASCs (4 × 10^6^).
• CM group (4): biomaterial implant containing TNF (25 ng/μL) (Gibco^®^) and CM.

Note that all contralateral limbs were used as a control.

For the surgical procedure, each rat was placed in lateral decubitus such that the lateral side of one hindlimb could be operated on. Once the sciatic nerve was exposed, it was dissected with microsurgical instruments (Figures 1B,C). After dissection, the biomaterial implant was placed in a cuff around the sciatic nerve using loose stitches of 6/0 non-absorbable suture material (nylon, Ethilon^®^) (Figures 1D,E). In no case did either the biomaterial or the suture compress or mechanically injure the nerve. To prevent displacement of the cuff, it was secured to an adjacent muscle plane using a loose stitch of the same suture material (Figure 1F).

Rats were maintained for 3 weeks following euthanasia with Pentobarbital (Dolethal^®^). Immediately sciatic nerve tissues were harvested using sterile techniques and stored in a phosphate buffered saline (PBS, Sigma^®^) with 1% (v/v) penicillin-streptomycin (Hyclone^®^).

#### Functional assessment of nerve regeneration: sciatic functional index

Walking track analysis was performed at 3 weeks after surgery, and assessment of the functional performance of the sciatic nerve was based on the Sciatic Functional Index (SFI) (Lee et al., 2015).

The SFI was calculated according to the equation:


$$S\,F\,I=-38.3\,\left(\frac{E\,P\,L-N\,P\,L}{N\,P\,L}\right)+109.5\,\left(\frac{E\,T\,S-N\,T\,S}{N\,T\,S}\right)+13.3$$

$$\left(\frac{E\,I\,T\,S-N\,I\,T\,S}{N\,I\,T\,S}\right)-8.8$$

Where print length (PL) is the length between the third toe and heel, the length between the first and fifth toes [toe spread (TS)] was calculated on the experimental side (E) and the contralateral normal side (N) in each rat, as well as the second and fourth toe spread [intermediary TS (ITS)]. The SFI scale runs from −100 to 0, where 0 indicates normal nerve activity and −100 indicates complete dysfunction. SFIs were evaluated for each experimental group.

#### Histology

In order to perform the histomorphological analysis of the nerves, samples were fixed in 10% neutral formalin and then dehydrated in a graduated ethanol series before being embedded in paraffin wax. Consecutive 8 μm sections were then cut from the paraffin blocks. The sections were deparaffinized and stained with hematoxylin and eosin (Sigma^®^) to assess the general morphology under an optical microscope (Nikon^®^).

The semi-fine sections obtained (0.5 μm) were stained with toluidine blue 1% (Panreac Química^®^) and observed in a Nikon^®^ E600 optical microscope.

#### Transmission electron microscopy

Nerve morphology was analyzed using Transmission electron microscopy (TEM). For this purpose, cell samples from all experimental rat groups were harvested 3 weeks after surgery and fixed overnight in 2.5% glutaraldehyde in 0.1 PBS. Samples were then fixed a second time, overnight, using 2% osmium tetroxide (TAAB Laboratories^®^) in PBS. Samples were then dehydrated in graded aqueous ethanol solutions and washed in propylene oxide (SERVA Electrophoresis GmbH^®^), finally, samples were infiltrated with epoxy resin and polymerized. The resulting blocks were cut into ultrafine sections (60 nm) using a diamond knife (Diatome^®^) under an LKB Ultrotome V microscope (Bromma^®^). Sections were then observed with a transmission electron microscope (JEM-1010 JEOL^®^) using a double contrast method (uranyl and plumb citrate). Images of 10 random fields were captured foreach section and hystomorphometric analysis was performed using NIS Elements microscope imaging software (Nikon^®^).

### Statistical analysis

Statistical analysis was performed using IBM SPSS Statistics 17. Significant differences between groups were determined using a one-way ANOVA followed by Tukey’s *post hoc* analysis. Results with *p* ≤ 0.05 were considered statistically significant. The quantitative results shown represent the mean ± SD of three independent experiments.

## Results

### Adipose tissue derived-mesenchymal stem cells and CM promote sciatic nerve regeneration after TNF-induced demyelination *in vivo*

TNF-treated nerves were compared to those with either ASCs or CM to examine their regenerative role in sciatic nerve.

**FIGURE 2:**
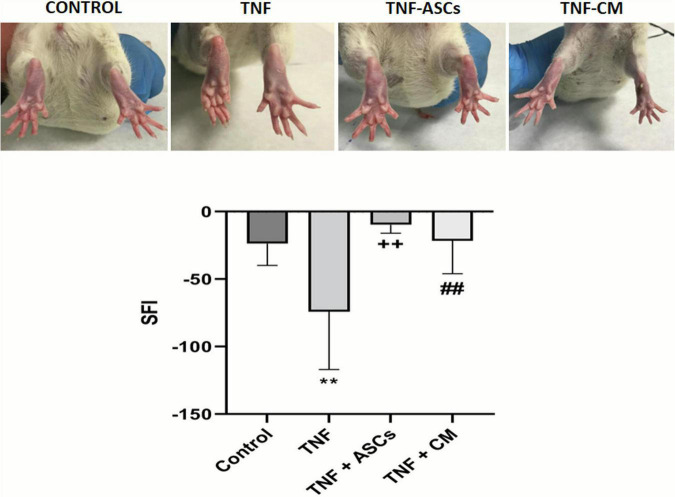
Post-operative appearance of rat limbs (upper 4 images): the different biomaterial implants have a clear effect on digit flexure with TNF treated rats showing severely contracted digits. Recovery of sciatic nerve function (graph): Comparison of SFI outcomes between groups: ** (*p* ≤ 0.01) compared with the control group; ++ (*p* ≤ 0.01), compared to the TNF group; ## (*p* ≤ 0.01) compared to the TNF group. The results are expressed as the mean ± SD of three independent experiments.

Experimental groups of rats received surgical implants to their sciatic nerves. Implants contained either no treatment (biomaterial control, group 1) or one of the 3 following treatments: TNF (group 2), TNF+ASCs (group 3) and TNF+CM (group 4). Walking track analysis was used to assess rats’ sciatic nerve function pre-operatively and 3 weeks post operatively. During this analysis, rats were allowed to walk on a blank piece of paper and plantar measurements were taken. This enabled us to generate pre- and post-operative SFIs for each rat. Before surgery, SFI values for rats in all groups were close to zero indicating normal functioning of the limbs. Post-operatively rats in groups 1, 3, and 4 were able to walk completely normally and barely any alteration to the operated limb was observed (Supplementary Videos 1, 3, 4). However, rats in group 2 (TNF treatment) appeared to present some muscle atrophy in their operated limb and did not place any weight on it when walking, keeping this limb curled up (Supplementary Video 2). In Figure 2, the digit positions for rats in all experimental groups are compared. Rats in the control group present with normal digit positions while those in group 2 (TNF group) present very abnormal digit positioning—digits are in fact, shrunken and curled up. The rats in which TNF inflammation was treated with ASCs or CM (groups 3 and 4 respectively) mostly present normal digit positions, in fact, digits look very similar to those of the control rats and to digits on the unoperated collateral limb. Looking in detail at the post-operative SFI’s for each experimental group: group 1 (control) had a mean value of −23.6; group 2 (TNF) recorded a mean value of −74.09; group 3 (ASCs) had a mean value of −9.7; and group 4 (CM) had a mean value of −21.5. The mean SFI’s for groups 3 and 4 are very similar, and slightly better, than that recorded for the control group (Figure 2). These results indicate that the functional recovery of the limb in the rats treated with either ASCs or CM is almost complete. Further analysis showed that only results for group 2 (TNF) were statistically significantly different from those of other groups: TNF with biomaterial, *p* = 0.0089, group 3 (TNF+ASCs), *p* = 0.0077, group 4 (TNF+CM), *p* = 0.0097.

### Morphological and morphometric analysis of regenerated sciatic nerves

**FIGURE 3:**
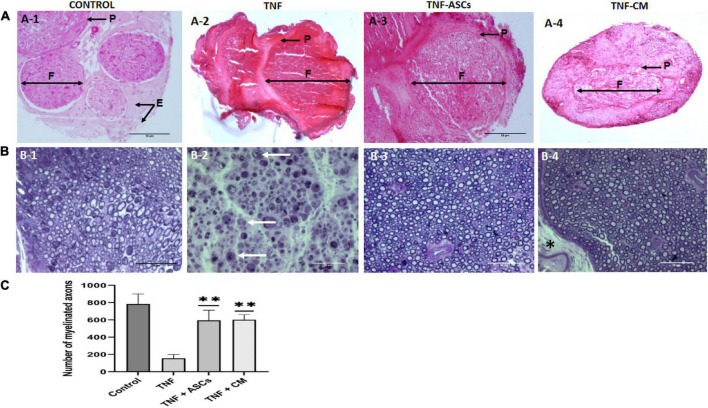
Rat sciatic nerve Histology. Representative images of hematoxylin-eosin staining (upper images). **(A)** From panels **(A-1–4)** tissue from control rats (biomaterial only); tissue from rats given biomaterial containing TNF; tissue from rats given biomaterial containing TNF+ACS; and tissue from rats given biomaterial containing TNF+CM. Images showing the nerve fascicles (F) grouped in bundles surrounded by perineurium (P) and epineurium (E). Scale bar: 20 μm. **(B)** Semi-thin slices of transverse sections of rat sciatic nerve stained with toluidine blue (lower images). From panels **(B-1–4)** normal tissue from the control group; tissue from the TNF-group axons show clear signs of degeneration and there is myelin degradation. Tissue from the ASC and CM groups both show signs of regenerated nerve fibers surrounded by newly formed myelin sheaths. The thick epineurium layer is marked with an asterisk (*). White arrows indicate degenerated myelin remnants. Scale bar: 50 μm. **(C)** Quantification of number of axons and comparation for all four groups. The results are expressed as the mean ± SD of three independent experiments ^**^p ≤ 0.01.

Nerve tissues harvested from the euthanized experimental rats were processed for histological analysis. Since the biomaterial was wrapped around the nerve no suture was needed, thus there were no possible effects to the nerve resulting from suturing. Sections stained with hematoxylin-eosin were used to assess the general histology of nerve tissues using optical microscopy. Figures 3A1–4 shows the general morphology of the nerve tissues collected from rats in each treatment group and controls. In a normal, non-damaged sciatic nerve, we would expect to see certain distinct structures. Myelinated nerve fibers run along the length of the sciatic nerve and the myelin sheath of each one is wrapped in a protective layer of connective tissue called the endoneurium. The nerve fibers themselves are bundled into groups called nerve fascicles (F) that are also wrapped in connective tissue, the perineurium (P). The numerous fascicles are, in turn, bundled together and wrapped in a further layer of connective tissue, known as the epineurium (E). Nerve tissues from the biomaterial control group of rats appeared normal (Figure 3A1). In contrast, nerve tissue from the TNF-treated group showed significant abnormalities, specifically, fascicles could not be identified due to the fact that the endo-, peri- and epineurium layers had disappeared; there was also evidence of demyelination (Figure 3A2). In addition, staining was more intense, and nerves lacked integrity compared to the biomaterial control group. For the groups that had received either ASCs or CM in addition to TNF, nerve tissues from both groups showed signs of degeneration in that the nerve fascicles had lost their original degree of organization (Figures 3A3,4). However, there was some indication of nerve regeneration. Of particular interest, samples from group 3 (TNF+ASCs) show the formation of new fascicles surrounded by a layer of perineurium (Figure 3A3).

**FIGURE 4:**
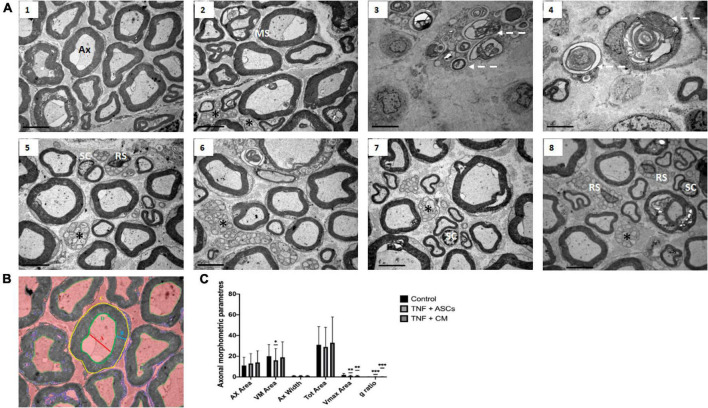
Rat sciatic nerve ultrastructure: **(A)** Representative images of transmission electron micrographs of rat sciatic nerves. (1,2) Control group showing normal axons (Ax) surrounded by a myelin sheath (MS). (3,4) TNF-treated group showing degenerated axons in (dotted white arrows) surrounded by remnants of degenerated myelin sheaths. (5,6) TNF+ASCs group showing unmyelinated axons (asterisks), myelinated Schwann cells (SC) and Remak Schwann cells (RS). (7,8) TNF+CM group showing enlarged Schwann cells (SC) surrounding some of the axons. Scale: 1 μm. **(B)** Axon parameters calculated for the *in vivo* rat model. A: axon internal diameter (AX width); B: myelin sheath width (VMax Area); C: total axon area (Tot Area); D: axon area (AX Area); E: g ratio = {(A/2)/[B+(A/2)]}. **(C)** Histograms comparing axon parameters for the three different groups (Control, TNF+ASCs, TNF+CM). We were not able to obtain any meaningful measurements of axon parameters for the TNF group since the nerves in these samples were unstructured and the relevant parameters could not be clearly identified. The results of morphometric analyses are expressed as the mean ± SD of three independent experiments and we analyzed 10 separate images for each experimental group were analyzed. *p ≤ 0.05 versus control group, ^**^p ≤ 0.01 versus control group, ^***^p ≤ 0.001 versus control group.

Sections stained with toluidine blue were used to specifically examine myelin, see Figures 3B1–4. In samples from the biomaterial control group, the bright blue staining of myelin makes it possible to distinguish the myelinated axons, in addition, nerve fascicles, and connective tissues (endo-, peri- and epineurium) are clearly visible. Samples from groups 3 and 4 (TNF+ASCs and TNF+CM) look very similar to those from the biomaterial control group with fully myelinated axons and a thick epineurium layer (Figure 3B4 indicated by an asterisk). However, for group 2 (Figure 3B2, TNF only), complete nerve degeneration can be observed. Not only is it impossible to identify nerve fascicles, because the endo- and perineurium have been destroyed, but there is also clear evidence of demyelination. In addition, there are signs of axonal degeneration and myelin disintegration (Wallerian degeneration). Number of axons were quantified and compared for all four groups (Figure 3C) and statistically significant differences were found between all groups except in the case of comparing groups 3 and 4 (TNF+ASCs and TNF+CM) (*p* = 0.847).

Transmission electron microscopy confirmed the structural and morphological changes observed using optical microscopy and revealed several others (Figure 4A). Nerves belonging to the biomaterial control group demonstrate the normal ultrastructure expected in the sciatic nerve and it is possible to identify numerous myelinated axons (Ax) surrounded by SCs. Unmyelinated axons (asterisks) are also present in smaller number (note that the myelin sheath (MS) is clearly distinguishable in Figures 4A1,2). Nerves from group 2 (TNF) show clear evidence of TNF induced neuropathy (Figures 4A3,4). Axons appear demyelinated and remnants of degenerated myelin are visible inside the axons (dotted white arrows). In contrast, inflamed nerve tissue treated with ASCs or CM (groups 3 and 4), show signs of intense nerve regeneration (Figures 4A5–8). Compared to nerve tissue from group 2 (TNF), there are far more myelinated axons (although not as many as seen in the biomaterial control group tissues, the epineurium. This structure can be recognized in the groups treated with ASCs and CM (Figures 4A3,4) while a clear degeneration of the structure in the groups treated with TNF is observed (Figure 4A2). In addition, a large number of unmyelinated axons are also present in these tissue samples, which may indicate the initiation of axonal regeneration. It should be noted that many of the SCs (labeled on Figures 4A5–8) seen in these samples appear enlarged, which, due to their well-known role in axonal regeneration, may provide some confirmation of nerve repair processes occurring in these samples.

In order to quantify the number of axons and assess their condition after undergoing the different experimental conditions, we measured a range of morphological parameters (Figure 4B). These parameters included: axon area (Ax Area); myelin sheath width (VM Area); axon internal diameter (Ax Width), total axon area (Tot Area) and myelin sheath internal area (Vmax Area). G-ratio which a very accurate ratio for measuring axonal myelination is, was also determined. It compares the inner axonal diameter to the total outside diameter. When axon g ratio reaches values near to 0.6, the conduction velocity is increased. The results are shown in Figure 4C. The analysis did not provide any measurable results with the TNF group since the nerves were unstructured and the parameters to be analyzed were not clearly identified. Comparing the other groups pairwise (Control, TNF-ASCs and TNF-CM), the only statistically significant differences were found for the variable VM Area (*p* = 0.017) comparing the biomaterial control and the TNF-ASCs group; the variable Vmax Area comparing the biomaterial control to the TNF-ASCs group and also the TNF-CM group where *p* ≤ 0.01 in both cases (Figure 4C) and g ratio comparing the biomaterial control to the TNF-ASCs group and also the TNF-CM group where *p* ≤ 0.001 in both cases.

### Conditioned medium induces myelination in TNF-stimulated nerves *ex vivo*

**FIGURE 5:**
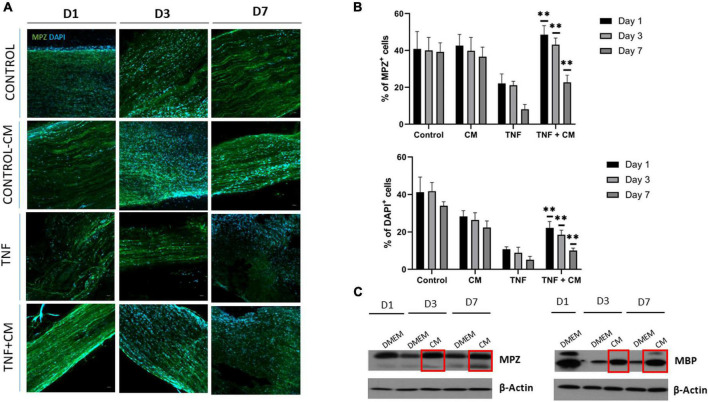
Sciatic nerves cultured with TNF or TNF and CM *ex vivo*. **(A)** Confocal microscopy. Images showing sciatic nerves cultured for 1, 3 and 7 days labeled with the myelin marker MPZ (and DAPI for DNA staining) in control conditions, in the presence of TNF, or TNF+CM. Scale bar: 50 μm. **(B)** Histogram comparing the number of cells as a percentage of DAPI-positive nuclei in the cultured nerves and the percentage of green-positive fluorescence representing the myelin protein MPZ (*p* ≤ 0.05, ^**^p ≤ 0.01 using ANOVA to compare TNF with TNF+CM treated samples). The results are expressed as the mean ± SD of four independent experiments. **(C)** Western blots showing the levels of myelin proteins MPZ and MBP in sciatic nerves cultured with DMEM and CM for 1, 3 and 7 days.

To examine the effect of CM directly on myelin sheaths, TNF-treated sciatic nerve segments were cultured in the presence or absence of CM. Confocal microscopy was used to analyze the myelin structural protein MPZ at 1, 3 and 7 days. Notably, less MPZ fluorescence (green) was observed in nerves cultured with TNF alone compared to those cultured with TNF and CM (Figures 5A,B), suggesting that CM increases myelin production or protects against demyelination. In addition, a significant decrease in DAPI staining was found in nerves cultured with TNF only compared to those cultured with TNF+CM (Figure 5B) implying that TNF actually reduced cell number.

To further explore the effect of CM on myelination, MPZ and MBP myelin protein levels were analyzed by western blotting at 1, 3 and 7 days. Results showed increased levels of the structural proteins MPZ and MBP after 3 days of CM-treatment (Figure 5C). This confirms that CM is able to upregulate the expression of myelin-related proteins or to suppress myelin degradation.

### Conditioned medium upregulates the expression of *Krox-20*, *Mpz* and *Periaxin* and downregulates the expression of *c-Jun* in nerves *ex vivo*

**FIGURE 6:**
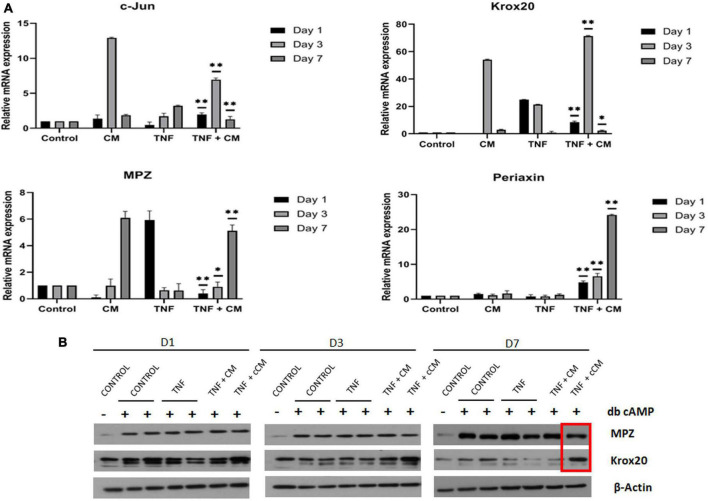
**(A)** CM induces myelination. Expression of myelin related transcription factors (*c-Jun*, and *Krox-20*) and genes encoding structural proteins (MPZ and Periaxin) in sciatic nerves inflamed with TNF (25 ng/mL) and treated with CM for 1, 3 and 7 days. The results are expressed as the mean ± SD of four independent experiments. *p ≤ 0.05, ^**^p ≤ 0.01 using ANOVA comparing TNF with TNF-CM treated samples. **(B)** TNF does not induce demyelination in *in vitro*-myelinated Schwann cells. Western blots showing levels of Krox-20 and MPZ myelin-related proteins in Schwann cells (SCs) inflamed with TNF (25 ng/mL) at 1, 3 and 7 days of culture. Technical duplicates are shown in the western blot.

The effects of CM on the regulation of the transcription factors *Krox-20* and *c-Jun* as well as the expression of the myelin related proteins *Mpz* and *Periaxin* were evaluated using qPCR. Sciatic nerve explants cultured in the experimental conditions previously described (see section “*Ex vivo* model of sciatic nerve damage”) were collected after 1, 3 and 7 days. After 7 days in culture, expression of *Mpz* and *Periaxin* was significantly higher (*p* ≤ 0.01) in nerve segments cultured in the presence of TNF and CM compared to those cultured with TNF alone. In addition, *c-Jun* was significantly downregulated in samples cultured with TNF alone in comparison to nerves treated with TNF and CM after 7 days in culture, (*p* ≤ 0.01) and *Krox-20* was dramatically upregulated in TNF+CM samples after 3 days of treatment (Figure 6A).

### TNF does not induce loss of myelin protein expression in *in vitro*-myelinated Schwann cells

To study the molecular mechanisms of TNF-induced demyelination observed in nerves, an *in vitro* Schwann cell (SC) myelination assay was performed. SCs were isolated and purified following db-cAMP treatment, a cell-permeable and non-hydrolysable cAMP analog. SCs were then switched to a starvation medium (without db-cAMP), which led to a clear decrease in myelin marker levels, showing that this model could be used to mimic demyelination *in vitro*. Results also showed that, 3 days after the addition of db-cAMP, there was an increase in the levels of both *Krox-20* transcription factor and myelin protein zero (MPZ) myelin structural protein (Figure 6B).

We analyzed whether TNF could induce demyelination in purified *in-vitro* myelinated SCs (i.e., in the absence of other cells like neurons or immune cells such as macrophages normally recruited by SCs after nerve damage) and the effect of CM on TNF-stimulated SCs. To make an initial assessment of TNF-induced demyelination in this model, we measured levels of Krox-20 and MPZ, at 1, 3 and 7 days after TNF addition. We did not observe changes in the protein levels at day 1 and 3, however, Krox-20 was slightly decreased after 7 days except in TNF and concentrated CM samples (Figure 6B). Surprisingly, MPZ levels were increased in all experimental conditions after 7 days. This suggests that, in purified SCs culture, in the absence of other type of cells such as macrophages TNF does not immediately induce demyelination. After 7 days, *in vitro*-myelinated SCs showed increased levels of Krox-20 protein when TNF and concentrated CM were added to cell culture. No changes were observed at days 1 and 3.

## Discussion

This study explores the effects of two potential regenerative therapies for peripheral nerve regeneration: the use of adipose tissue derived-mesenchymal stem cells (ASCs) and ASC-derived conditioned medium (CM) after TNF-induced nerve injury. To further explore the nerve regeneration potential of ASCs and CM we used both *ex vivo* and *in vivo* models of nerve damage, using either TNF-treated rat sciatic nerves or biomaterial implants containing TNF to induce local nerve damage, respectively. Our data suggest that ASCs and CM both have substantial neuroprotective and axonogenic effects, promoting the sciatic nerve function after TNF-induced nerve injury. Findings from our *in vivo* and *ex vivo* model showed that both ASCs and CM aided the regeneration of axonal myelin sheaths and the remodeling of peripheral nerve morphology. These are promising results for the alternative treatment of degenerative neuropathies with an inflammatory origin.

Myelinating SCs are the primary glial cells of the peripheral nervous system (PNS). Disruption of these cells occurs in response to a variety of clinical circumstances, including exposure to inflammatory factors such as TNF and can lead to myelin breakdown and neuropathy. Here, we used purified SCs to induce myelination *in vitro* (Sobue and Pleasure, 1984) and investigate TNF-induced myelin degradation in SCs. SCs cultured with TNF showed no observable changes in levels of key myelin-related proteins until 3 days after SCs inflammation with TNF. Thus, contrary to data documented in other studies (Wagner and Myers, 1996b; Leung and Cahill, 2010; Lee et al., 2018), our results suggest no direct effect of TNF on SC demyelination caused by TNF. In addition, culturing TNF-stimulated SCs with concentrated CM only produced increase levels of MPZ and Krox-20 after 3 days which suggests that SC activation is impeded without the presence of other activated cells (e.g., neurons, immune cells).

Nerve regeneration is possible due to the plasticity of SCs, specifically their ability to revert to an immature phenotype known as repair SCs and this process has been observed in response to physical nerve injury and in peripheral neuropathies (Parkinson et al., 2008; Jessen et al., 2015; Jessen and Mirsky, 2019). After nerve damage, SCs respond to axon signals to begin the nerve regeneration by first dedifferentiating and proliferating, followed by redifferentiation and remyelination of newly formed axons (Harrisingh et al., 2004). SCs are effectively reprogrammed, first by downregulation of myelin-related genes and the activation of a set of repair-supportive features, including trophic factors, and cytokines followed by induction of autophagy and macrophage recruitment to remove myelin debris, and finally, formation of regeneration tracks, known as Bungner’s bands, which guide newly formed axons (Jessen and Mirsky, 2000).

The transcription factor *c-Jun* is known to be a key regulator of SC plasticity; it prevents the activation of myelin genes by inhibiting the transcription factor *Krox*-20. In injured nerves, *c-Jun* also induces dedifferentiation of myelinating SCs to their immature form required for nerve regeneration. Hence, *c-Jun* and *Krox-20* show a cross-antagonistic functional relationship; *c-Jun* therefore negatively regulates the myelinating SC phenotype, representing a signal that functionally stands in opposition to the pro-myelin transcription factors such as *Krox-20* (Arthur-Farraj et al., 2012; Roberts et al., 2017). Our results confirmed *c-Jun* upregulation after TNF-mediated nerve damage, whereas *Krox-20* was downregulated. We observed an increase in myelin-related genes and their corresponding proteins including Krox-20, MPZ, MBP and Periaxin (Morris et al., 1972; Jessen and Mirsky, 2019) in the presence of CM (Chen et al., 2007; Arthur-Farraj et al., 2017). TEM images of TNF-treated nerves with either ASCs or CM show the presence of the repair SCs, as well as myelinating and Remak SCs (Figures 4A1–8).

It has been established that *c-Jun* is one of the earliest transcription factors to be activated after nerve damage, and its expression increases in the hours after damage has occurred (Gomez-Sanchez et al., 2017). However, we observed that in TNF-stimulated nerves treated with CM, *c-Jun* mRNA levels were downregulated. *Krox-20* on the other hand, must be upregulated in SCs to express myelin-related proteins and begin the process of rebuilding myelin sheaths. Thus, the finding that CM appears to downregulate *c-Jun* while upregulating *Krox-20* and other myelin-related proteins show for the first time an important role of CM in nerve regeneration.

In our *in vivo* model, we observed that, over the first 5–7 days following TNF stimulation, the SCs themselves break down 40–50% of the myelin in the initial phase of myelin clearance in Wallerian degeneration (Perry et al., 1995). This tends to point to the key role of macrophages in myelin breakdown; as observed by other authors (Hirata and Kawabuchi, 2002; Vargas et al., 2010). These immune cells assist Wallerian degeneration infiltrating damaged nerves and phagocytosing myelin debris. Our TEM images show the destruction of TNF-treated nerves by SCs and immune cells (Figure 4). Importantly, we discovered that myelin sheaths of TNF-mediated damaged nerves recovered typical nerve morphology in rats treated with CM.

Our data suggest a potential therapeutic benefit of ASCs and CM for the treatment of neuropathy. The efficacy of these treatments may lie in the paracrine effects described in several studies showing that MSCs contribute to the regeneration of damaged tissue by releasing a large number of trophic factors and microvesicles that favor axonal regeneration and myelination of nerve fibers (Cho et al., 2009; Rivera and Aigner, 2012; Tomita et al., 2012). Our results appear to support the paracrine hypothesis since axonal quantification in samples from our *ex vivo* study show that treating TNF-stimulated nerves with ASCs or CM induced a recovery in the number of axons (Cho et al., 2009; Rivera and Aigner, 2012; Tomita et al., 2012).

In summary, our findings confirm clear evidence of the beneficial effects of ASCs and particularly CM in nerve regeneration using rat as *in vivo* model. In the presence of CM, *c-Jun* was downregulated while *Krox-20* was upregulated, as well as other myelin related proteins including MBP; MPZ.

Our model for nerve injury were based on TNF-induced nerve damage and although TNF did not have a direct effect on SCs *in vitro*, in sciatic nerves explants and in our *in vivo* model TNF induced demyelination by activating and increasing macrophage recruitment, respectively. Morphological analysis of these nerves shows that ASCs and CM were able to both protect sciatic nerves from demyelination and start the nerve regeneration process. TEM images show the presence of great number of immature SCs in samples treated with ASCs and CM. However, the present study had some limitations, such as lack of a neurophysiological test to evaluate the nerve regeneration and the capacity of conduction in the treated nerves. Furthermore, it is important confirm that ASCs and CM modulate the macrophage phenotype and may contribute to regeneration in sciatic nerve. Nonetheless, more research is needed to determine the underlying mechanisms by which ASCs and CM are able to induce the regeneration of damaged nerves.

## Data availability statement

The original contributions presented in this study are included in the article/Supplementary material, further inquiries can be directed to the corresponding author/s.

## Ethics statement

All procedures were performed following the ethical guidelines of the Biosafety and Welfare Committee of the University of León and Junta de Castilla y León (OEBA).

## Author contributions

VV-S had full access to all the data in the study and was responsible for the integrity of the data and the accuracy of the data analysis. VV-S, EG-C, MP-I, and AW contributed to conception and design of the study. EG-C, MG-F, MR-D, and MP-I performed the data acquisition. All authors performed analysis and interpretation of the data, involved in drafting the article or revising it critically for major intellectual content, and approved the final version to be published.
